# Tibial Lengthening along Submuscular Plate with Simultaneous Acute Tibial Deformity Correction by High-Energy Osteotomy: A Comparative Study

**DOI:** 10.3390/jcm11185478

**Published:** 2022-09-18

**Authors:** Kuei-Yu Liu, Kuan-Wen Wu, Chia-Che Lee, Sheng-Chieh Lin, Ken N. Kuo, Ting-Ming Wang

**Affiliations:** 1 Department of Medical Education, National Taiwan University Hospital, Taipei 100, Taiwan; 2 Department of Orthopaedic Surgery, National Taiwan University Hospital, Taipei 100, Taiwan; 3 Department of Orthopaedic Surgery, Chung Shan Medical University Hospital, Taichung 402, Taiwan; 4 Institute of Medicine, Chung Shan Medical University, Taichung 402, Taiwan; 5 Cochrane Taiwan, Taipei Medical University, Taipei 110, Taiwan; 6 Department of Orthopaedic Surgery, College of Medicine, National Taiwan University, Taipei 100, Taiwan

**Keywords:** submuscular plating, high-energy osteotomy, limb lengthening, angular deformity

## Abstract

Submuscular plating and osteotomy using power saw have shown the benefits in certain situations of limb lengthening. However, no previous studies combining both procedures have been conducted for acute tibial deformity correction and limb lengthening. Nineteen cases were enrolled in this study. Ten patients received tibial lengthening with acute knee angular deformity correction using high-energy osteotomy (Group 1), and nine patients received tibial lengthening only with osteotomy using multiple drills and osteotome (Group 2). Radiographic parameters retrieved before and after the operation included leg-length discrepancy, tibial length, length gained, mechanical lateral distal femoral angle (mLDFA), medial proximal tibial angle (MPTA), and mechanical axis deviation (MAD). There were significant differences between groups in terms of external fixator index (EFI) (*p* = 0.013) and healing index (HI) (*p* = 0.014), but no significance in the length gained (*p* = 0.356). The latest postoperative mLDFA (*p* = 0.315), MPTA (*p* = 0.497), and MAD (*p* = 0.211) of Group 1 were not distinguishable from Group 2. The functional outcomes were excellent, and there were no permanent complications. Despite showing a longer healing time, this alternative lengthening procedure which combines fixator-assisted plate lengthening in the tibia with simultaneous surgical intervention of acute tibial deformity correction using an oscillating saw is appropriate for patients with leg-length discrepancy and angular deformity of the tibia.

## 1. Introduction

Distraction osteogenesis with an external fixator is commonly performed for correcting limb-length discrepancies [1,2]. However, several complications have been reported due to the long duration of external fixator application [2,3]. Lengthening along an intramedullary nail was initially proposed in order to solve common complications of external fixators including pin-tract infection, limited joint range of motion, and muscle contracture [2,3]. A reduction in the duration of external fixation has been reported with minor inconvenience and less time for regaining normal motion of the joints, which leads to lower incidence of muscle contracture and joint instability [4,5]. Nevertheless, the procedure may not be recommended under certain circumstances, such as in preadolescents [6]. Submuscular plating was, thus, proposed, and the result outperformed the lengthening over the nail in a wide variety of age groups with knee angular deformity, physeal injury, or a narrow medullary canal [6,7,8]. This alternative procedure helps preserve the endosteum and maintain the angular stability [9,10]. It can also shorten the duration of the fixator without disturbance of the regeneration of distraction callus [7,11]. For major complications, deep intramedullary infection has been reported in studies of using intramedullary nails because of the risk of combining fixation pins with intramedullary nails at the same time [5,8,12]. However, small case series reports using submuscular locking plates showed no cases of deep infection [8,11].

Both lengthening along the nail and submuscular locking plating were also demonstrated as efficient methods for correction of distal femoral valgus deformity, resulting in a similar final mechanical axis deviation correction [13]. Treatment with the Taylor spatial frame was excellent at correcting lower-extremity long bone deformities, and Ilizarov frames were superior in straightforward lengthening of long bones [14]. Nevertheless, techniques, outcomes, and complications have not been well clarified in fixator-assisted plate lengthening with simultaneous correction of knee angular deformities.

For the techniques of osteotomy in limb lengthening, studies were often performed with low-energy osteotomy including multiple drill holes and osteotomes [7,8,15]. This technique preserves the medullary circulatory system, leading to optimal bone formation [16]. When perfect bony surfaces in closing wedge osteotomies for correcting joint angular deformities are required, power saw osteotomy surpasses the conventional method [16,17]. This method of high-energy osteotomy formed nice bone-to-bone contact that enhances bone formation, yet it had the potential to cause delayed consolidation in animal studies [16,17]. So far, no previous studies combining submuscular plating and high-energy osteotomy have been conducted for acute tibial deformity correction and limb lengthening

Therefore, in this retrospective case series study, we describe a technique of tibial lengthening along plate with simultaneous surgical intervention of acute tibial deformity correction. Our goal was to elaborate the outcome of the procedure and the effect caused by high-energy osteotomy, along with a comparison to straight tibial lengthening using the standard osteotomy technique.

## 2. Materials and Methods

### 2.1. Patients

We reviewed the cases who underwent tibial lengthening with submuscular plating from 2015 to 2019 at our institution. The inclusion criteria were tibial lengthening in patients with knee angular deformity requiring a metaphyseal osteotomy and a minimum of 2 years follow-up. Cases with combined femoral lengthening or a follow-up less than 2 years were excluded. Ten males and nine females were included in this retrospective study. Among 19 patients, 10 received tibial lengthening with knee angular deformity correction using an oscillating saw (Group 1), and nine received tibial lengthening without angular deformity correction using multiple drill holes and an osteotome (Group 2). Among all 10 cases who required knee angular deformity correction, there were varus deformity corrections on four limbs and valgus deformity corrections on six limbs. All cases were performed by the senior author (T.-M. Wang).

The average age at operation was 14.1 years (range, 7–35 years). The average postoperative follow-up period was 55.1 months (range, 28–89 months). The mean preoperative body mass index was 21.1 kg/m^2^ (range, 13.6–32.3 kg/m^2^). The mean preoperative leg-length discrepancy was 4.4 cm (range, 2.3–7.1 cm). Demographic features of all 19 patients included in Groups 1 and 2 are listed in Table 1. There were no significant differences between groups in parameters including age at operation, follow-up period, gender, operated side, leg-length discrepancy, and body mass index.

The etiologies of the deformity included fibular hemimelia (*n* = 6), idiopathic leg-length discrepancy (*n* = 4), sequelae of infection (*n* = 2), X-linked hypophosphatemic rickets (*n* = 2), congenital pseudoarthrosis of tibia (*n* = 2), neglected developmental dysplasia of the hip (*n* = 1), Ollier disease (*n* = 1), and neurofibromatosis (*n* = 1). Among all 19 patients, monolateral fixators (Limb Reconstruction System; Orthofix SRL, Verona, Italy), Ilizarov apparatus (Smith and Nephew, Memphis, TN, USA), and Taylor spatial frames (Smith and Nephew, Memphis, TN, USA) were applied to one, 11, and seven patients, respectively. The patient authorization forms for medical record use and academic research purposes were obtained from the patients or guardians. The study was approved by the Institutional Review Board of National Taiwan University Hospital.

### 2.2. Operative Technique

For those patients with knee angular deformities, a proximal tibia wedge resection was carried out with an oscillating saw for alignment correction. While making a closing wedge osteotomy for deformity correction, sub-periosteal dissection was carefully performed around the tibia osteotomy site. Furthermore, the periosteum and nearby vasculature were protected by gentle retraction. For those without angular deformity, multiple drill holes were carried out, followed by using an osteotome to finish the osteotomy. The locking plate (Synthes, Oberdorf, Switzerland) was then inserted into the submuscular space in both groups of patients. The plate was fixed at the proximal lateral side of the tibia with three or four screws, and the epiphyseal plate was protected. It was long enough for three or four screw lengths at the distal portion of the plate for the expected lengthening. In order to stabilize the plate and maintain the alignment during the procedure, temporary insertion of distal screws was performed. Subsequently, an external fixator was applied for lengthening. Then, the distal temporary screws were removed.

The rate for distraction was about 1 mm per day starting 7 to 10 days postoperatively, which could be adjusted according to the patient’s age and tolerance. The patients had fortnightly outpatient visits for assessment and minor adjustments of the external fixators if necessary.

Once the expected lengthening was achieved, the external fixator was removed, and additional screws were inserted into the distal portion of the locking plate. The circulation, sensation, motor function, and complications were monitored at follow-up clinics. Consolidation of at least three cortices was considered as healed lengthening. Patients were encouraged to start partial weightbearing at an appropriate time depending on the individual condition before progressing to full weightbearing. Physical therapy was ordered for joint range of motion. The locking plate could be removed after full consolidation of cortices and full weightbearing.

### 2.3. Radiographic Examination

**Figure 1:**
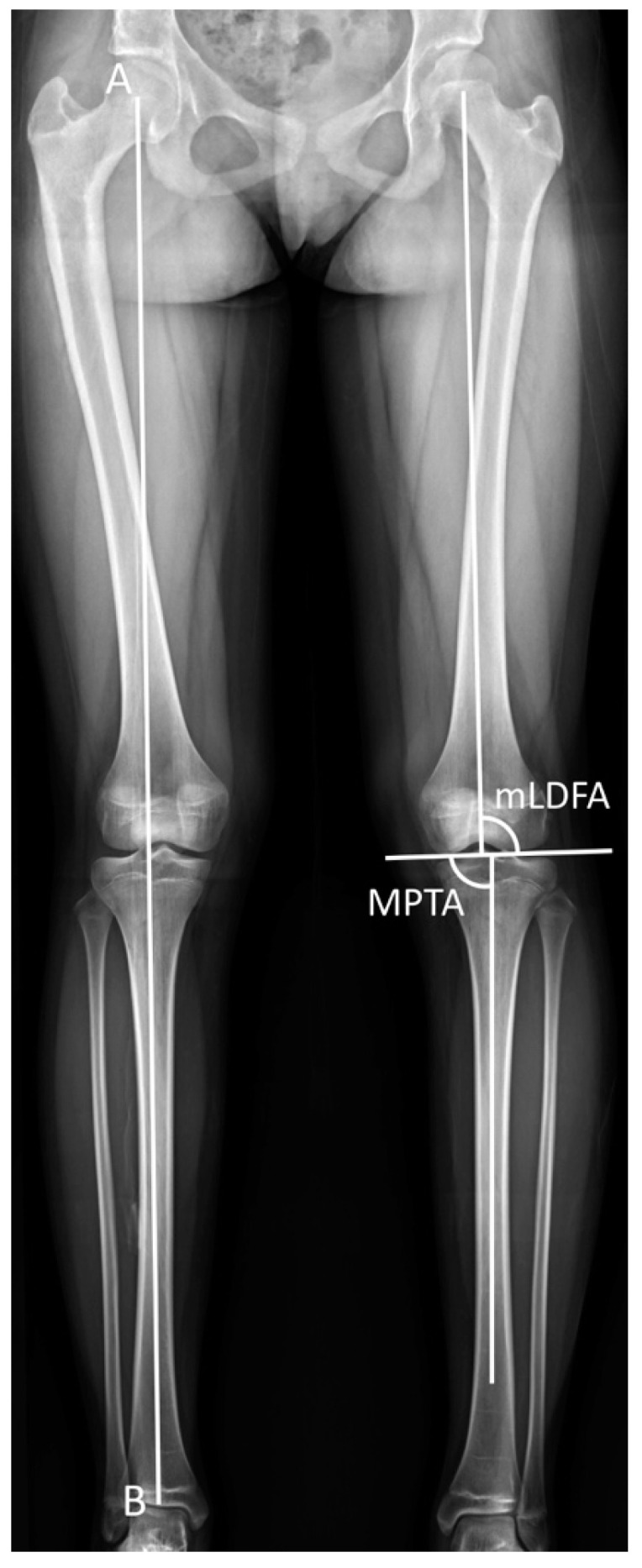
Radiological measurements. The superolateral angle between the mechanical axis of the femur and the joint surface of the femoral condyle was defined as the mechanical lateral distal femoral angle (mLDFA). The inferomedial angle between the anatomical axis of the tibia and the articular axis of the proximal tibia was labeled as the medial proximal tibial angle (MPTA). The mechanical axis deviation (MAD) was designated as the distance from the center of the knee to the mechanical axis of the lower leg. Mechanical axis = AB.

The scanogram was taken in a standing position with both knees fully extended and patellae facing forward. The tibia radiographs were obtained at each clinic visit. The radiographic parameters included leg-length discrepancy, tibial length, length gained, mechanical lateral distal femoral angle (mLDFA), medial proximal tibial angle (MPTA), and mechanical axis deviation (MAD) (Figure 1). The tibial length was measured from the tibial spine to the center of the tibial plafond. The length gained was defined as the increase in length of the operated limb. The mLDFA was defined as the lateral angle between the joint surface of the femoral condyle and the femoral mechanical axis, and the MPTA was defined as the medial angle between the articular axis of the proximal tibia and the tibial anatomical axis [18]. The distance from the center of the knee to the mechanical axis of the lower leg was labeled as the MAD [18]. The MAD was denoted as valgus or varus malalignment when the mechanical axis showed a lateral or medial deviation [18].

### 2.4. Statistical Analysis

The intra-rater and inter-rater reliabilities of the leg-length discrepancy, length gain, mLDFA, MPTA, and MAD in the reliability tests were analyzed by the intraclass correlation coefficient (ICC). Regarding the intra-rater reliability, data were measured repeatedly by the first author (K.-Y. Liu) at a 1-week interval. For inter-rater reliability, the above measurements were measured by two authors on separate occasions using the same method. All statistical analyses were performed using the Statistical Package for Social Sciences (SPSS) version 25.0 (IBM, Armonk, NY, USA). The intra-rater ICCs for leg-length discrepancy, length gain, mLDFA, MPTA, and MAD were 0.996, 0.997, 0.999, 0.999, and 0.997, respectively. The inter-rater ICCs were 0.992, 0.993, 0.997, 0.998, and 0.997, respectively.

The statistical analysis was performed using the Pearson chi-squared test and Mann–Whitney U test, determining the outcome differences among different groups and measurements. The statistical significance for all analyses was defined as *p* < 0.05.

## 3. Results

Demographic features of all 19 patients included in Groups 1 and 2 were listed in Table 1. The parameters including the age at operation, follow-up period, gender, operated side, leg-length discrepancy, and body mass index showed no significant difference between groups.

Clinical outcomes of the length gained, external fixation index, and healing index are enumerated in Table 2. The external fixation index (EFI) was defined as the duration between external fixator application and its removal, divided by the length gained. The healing index (HI) was labeled as the duration between external fixator application and appearance of at least three cortices, divided by the length gained.

Group 1 had a mean length gain of 4.5 cm (range, 1.6–6.7 cm), an average EFI of 22.1 days/cm (range, 12.4–30.6 days/cm), and a mean HI of 78.0 days/cm (range, 29.9–132.4 days/cm). Group 2 had an average length gain of 5.3 cm (range, 2.8–7.1 cm). The mean EFI and HI were 15.4 days/cm (range, 11.9–22.9 days/cm) and 42.7 days/cm (range, 26.5–102.5 days/cm), respectively. Overall, there were significant differences between groups in the EFI and HI but not the length gained.

**Figure 2:**
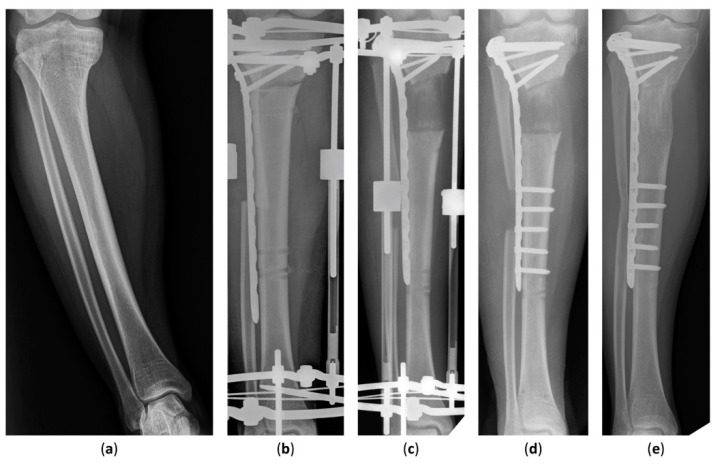
Anteroposterior radiographs of an 18-year-old girl who underwent fixator-assisted plating and acute angular correction for leg-length discrepancy. (**a**) A preoperative anteroposterior view of the tibia. (**b**) The procedure was conducted using an Ilizarov apparatus and a submuscular plate. (**c**) Satisfactory tibial lengthening and alignment were achieved. (**d**) The distal part of the submuscular plate was fixed at removal of the external fixator. (**e**) The distracted callus healing with good consolidation was seen in the latest radiograph 40 months after the operation.

**Figure 3:**
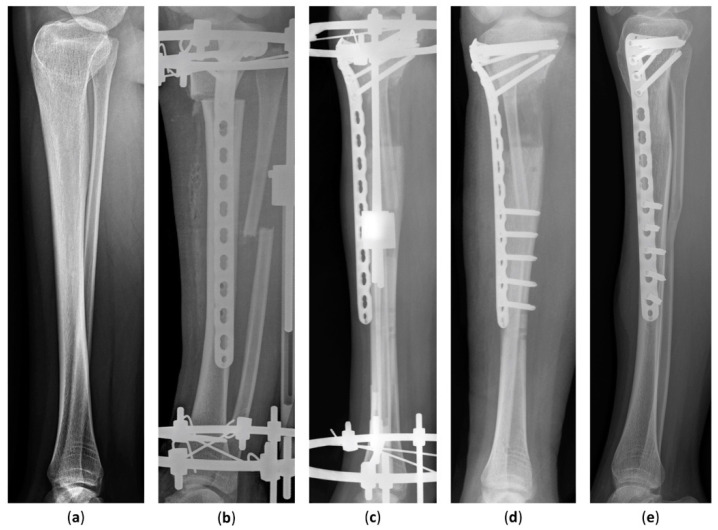
Lateral radiographs of an 18-year-old girl who underwent fixator-assisted plating and acute angular deformity correction for leg-length discrepancy. (**a**) A preoperative lateral view of the tibia. (**b**) The patient underwent tibial lengthening and deformity correction using an Ilizarov apparatus and a submuscular plate. (**c**) The distraction length and alignment were satisfied. (**d**) The distal part of the submuscular plate was fixed at removal of the external fixator. (**e**) Distraction osteogenesis was completed without complications 40 months after the procedure.

The latest postoperative radiographic measurements including the mLDFA, MPTA, and MAD are summarized in Table 3. The mean postoperative mLDFA was 85.0° (range, 79.5°–91.0°) in Group 1 and 86.5° (range, 80.0°–93.4°) in Group 2. The mean postoperative MPTA was 86.4° (range, 80.2°–93.5°) in Group 1 and 87.6° (range, 81.3°–93.5°) in Group 2. The postoperative MAD, the indicator of lower-limb malalignment, was 14.2 mm (range, 1.0–46.0 mm) in Group 1 and 6.7 mm (range, 3.0–13.0 mm) in Group 2. All in all, the postoperative mLDFA, MPTA, and MAD of Group 1 were not distinguishable from Group 2. The case of an 18-year-old girl with hypophosphatemic rickets who underwent acute angular correction and fixator-assisted lengthening is demonstrated in Figure 2 and Figure 3 at 40 months follow-up.

In this study, no procurvatum or recurvatum deformities were documented. There were no complications or plate failure in our series. There was no deep infection on our follow-up. Three patients who received tibial deformity correction experienced transient complications: one with drop foot, and two with knee and foot flexion contractures. All of them recovered the preoperative knee and ankle range of motion at the latest clinic visits after regular physical therapies.

## 4. Discussion

The purpose of this retrospective cohort study was to elucidate the effect of osteotomy using a high-energy power saw in bone growth and to ascertain the possible complications, as well as outcomes, after tibial lengthening along submuscular plate with simultaneous acute tibial deformity correction. In the current study, the HI and EFI of the operated limbs performed with high-energy osteotomy in Group 1 showed higher results and significantly longer time compared to Group 2 using multiple drills. However, there were no complications in Group 1 related to this longer healing time. With good correction of the deformity in Group 1, the final mLDFA, MPTA, and MAD of the operated limbs with tibial deformity correction were not distinguishable from Group 2. To the best of our knowledge, this is the report with longest follow-up and largest case numbers with respect to performing high-energy osteotomy for acute correction of knee angular deformity and fixator-assisted plating for tibial lengthening.

High- and lower-energy osteotomies differ mostly in the results of osteogenic potential [16]. Patients with tibial deformity in this study received osteotomies with oscillating saws instead of osteotome or multiple drill holes, which is commonly practiced [7,8,15,19,20]. Power saw osteotomies are often applied in closing wedge osteotomies that require perfect bony surfaces for good bone-to-bone contact, and the complication of thermal necrosis of the bone and soft tissue could happen throughout the procedure [16]. From the perspective of animal research, this method may result in decreased bridging vessels and delayed consolidation of cortices [17]. However, this conclusion lacks human studies. Since high-energy osteotomies lack appropriate surgical indications for non-correction patients, designing a study that completely matches patients with regard to the existence of joint deformity remains a difficulty. Unlike the power saw, multiple-drill osteotomy is a low-energy procedure that minimizes devascularization of the bone and periosteum [21,22]. Although multiple-drill osteotomy still induces short-term damage to the medullary vascularity, it has been recommended in distraction osteogenesis due to its ideal regenerative properties and minimal risk of thermal necrosis of bone ends [16,21,22,23]. In our study, we performed high-energy osteotomies on tibias which required acute deformity correction and used conventional multiple drill osteotomies on those without the need for angular correction. Although there was a delay in EFI and HI, results of limb alignment after the correction were outstanding.

So far, no tibial lengthening studies of submuscular plating with simultaneous surgical intervention of acute tibial deformity correction with an oscillating saw have been reported. On the basis of current knowledge, we combined external fixators and submuscular plates for tibial deformity correction [24,25,26]. Recent studies compared the types of external fixators in tibial deformity correction with or without simultaneous limb lengthening [24,25]. Birch et al. reported that the Ilizarov apparatus is better than the monolateral fixator since the configuration of the frame can be adjusted while correcting the knee angular deformity, while Rozbruch et al. advocated that the Taylor Spatial Frame can be applied in patients with limb-length discrepancy, postinfectious sequelae, or a poor soft-tissue envelope [24,25]. When comparing fixator-assisted plating or nailing in correction of the deformity, both methods developed accurate correction of distal femoral valgus deformity and showed similarities in the final knee range of motion [26]. As for tibial deformity, Hamada et al. performed osteogenesis and correction of tibial deformity with only the Taylor spatial frame [27]. We, therefore, performed a modified combination of the abovementioned techniques to correct the deformity in one stage and lengthen the tibia by gradual distraction. The outcomes of mLDFA, MPTA, and MAD demonstrated a satisfactory result. Considering the good functional results and low complication rates of this study, we believe that simultaneous acute tibial deformity correction by osteotomy using an oscillating saw and tibial lengthening with submuscular plating is a good alternative procedure for correcting knee angular deformities with leg-length discrepancy. There is no need for additional procedures based on our satisfactory radiographic outcomes. As pediatric patients with limb deformities are prone to behavioral problems compared to the adult patients, a lower chance of the second corrective surgery helps reduce their psychological effects [15].

In limb lengthening with external fixators, the complication rate rises along with the duration of external fixation including pin-tract infections [3,28]. Although fixation pins result in open tracts down into the bone, nearly all infected patients responded well to oral antibiotics and local pin-tract care in the previous studies [11,28,29]. Hamada et al. treated tibial deformity with merely external fixators, and the results showed a higher EFI compared to our technique which combined external fixators and submuscular plates [27]. We suggest that using a power saw for good bone-to-bone contact and inserting submuscular plates for adequate stability can help shorten the duration of external fixation [7,11,16]. Oh et al. reported peroneal nerve palsy or joint stiffness using a thick plate or short plate, respectively [11,20]. In our study, three cases with drop foot and restricted joint range of motion initially regained their preoperative mobility after rehabilitation at final follow-up. Therefore, we recommend selecting suitable plates individually and encouraging early rehabilitation to avoid limited knee and ankle range of motion.

Our study had limitations. First, the cases were not randomized or completely matched between groups with respect to the etiology. There were difficulties matching all parameters since our procedure was uncommon and the patient population was small. Second, the sample number was limited. However, it was a large sample size of a single-surgeon study in view of the rare eligible cases in nature. Furthermore, a single-surgeon study is expected to result in less inter-surgeon variability. Our study elaborates the results of a combined method of submuscular plating and acute tibial deformity correction with high-energy osteotomy, which is not commonly performed. Further evaluations with a larger patient number and a longer follow-up period are encouraged to clarify more details of the procedure.

## 5. Conclusions

To conclude, we demonstrated an appropriate alternative lengthening procedure which combines lengthening along submuscular plating with simultaneous surgical intervention of acute tibial deformity correction with an oscillating saw for patients with knee angular deformity and leg-length discrepancy. Although it increased the EFI and HI by nearly two times compared to the multiple drill osteotomy, the functional outcomes were excellent, and there was no nonunion.

## Figures and Tables

**Table 1:** Demographic features of the included patients.

	Group 1 (*n* = 10)	Group 2 (*n* = 9)	*p*-Value
Mean age at operation (years)	15.4 ± 7.5	12.7 ± 8.6	0.315
Mean follow-up (months)	53.0 ± 19.6	57.3 ± 23.5	0.720
Gender	Male 4; female 6	Male 6; female 3	0.245
Side involved	Left 4; right 6	Left 6; right 3	0.245
Mean leg-length discrepancy (cm)	4.6 ± 2.6	4.5 ± 1.6	0.447
Mean body mass index (kg/m^2^)	22.5 ± 6.9	19.5 ± 4.6	0.400

**Table 2:** The clinical outcomes of the included patients.

	Group 1 (*n* = 10)	Group 2 (*n* = 9)	*p*-Value
Mean length gained (cm)	4.5 ± 1.9	5.3 ± 1.4	0.356
Mean EFI (days/cm)	22.1 ± 4.4	15.4 ± 3.7	0.013 *
Mean HI (days/cm)	78.0 ± 36.5	42.7 ± 23.8	0.014 *

EFI, external fixation index; HI, healing index. * Statistical significance.

**Table 3:** The postoperative radiographic measurements at the latest outpatient visit.

	Group 1 (*n* = 10)	Group 2 (*n* = 9)	*p*-Value
Mean mLDFA (°)	85.0 ± 4.1	86.5 ± 4.1	0.315
Mean MPTA (°)	86.4 ± 3.6	87.6 ± 3.9	0.497
Mean MAD (mm)	14.2 ± 14.1	6.7 ± 3.2	0.211

mLDFA, mechanical lateral distal femoral angle; MPTA, medial proximal tibial angle; MAD, mechanical axis deviation.

## Data Availability

Not applicable.
